# Supplementary material for: Gallocin A, an Atypical Two-Peptide Bacteriocin with Intramolecular Disulfide Bonds Required for Activity
Source: Microbiol Spectr. 2023 Mar 23;11(2):e05085-22. doi: 10.1128/spectrum.05085-22 (PMC10100652; doi:10.1128/spectrum.05085-22)
Supplement: Supplemental file 1 — Fig. S1 to S7. Download spectrum.05085-22-s0001.pdf, PDF file, 13.5 MB [file spectrum.05085-22-s0001.pdf]

SUPPLEMENTARY FIGURE LEGENDS

Fig. S1. Gallocin A is active in a broad range of pH and heat-stable

A) Agar diffusion assay to test gallocin activity from supernatants of UCN34 WT and Δ*blp* at different pH against *SGM*, a gallocin A-sensitive bacterium. The initial supernatant from an overnight culture (left well) had a pH of 5.4. pH was then adjusted to 2-12 using HCl or NaOH. **B)** Supernatant was heated at 80°C for indicated times and tested as in (A). One experiment is shown here that was repeated twice independently.

Figure S2: Gallocin A spectrum of action

Agar diffusion assay using UCN34 WT and Δ*blp* supernatant against various bacterial species. One representative plate of three independent replicates is shown.

Fig. S3: LC-MS analysis of the two peptides constituting gallocin A indicating that both peptides contain a disulfide bridge.

Left Panel: Top – GllA2 structure, molecular formula and molecular weight (with disulfide bridge); Middle – Mass Spectrum showing the $MH_3^{+3}/3$ and $MH_4^{+4}/4$ masses observed for GllA2; Bottom – LC chromatogram showing the peak where GllA2 was detected. Right Panel: Top – GllA1 structure, molecular formula and molecular weight (with disulfide bridge); Middle – Mass Spectrum showing the $MH_2^{+2}/2$ and $MH_3^{+3}/3$ masses observed for GllA1; Bottom – LC chromatogram showing the peak where GllA1 was detected.

Fig. S4: Structural models of GllA1, GllA2 and GIP alone or in complex. All representations are colored with predicted lDDT from a score of 30% (red) to 100% (blue). The disulfide bond is visible in stick representation for GllA1 and GllA2.

Fig. S5: Characterization of 12 spontaneous mutants (RSM) resistant to gallocin A as compared to the parental sensitive strain *SGM*.

A) Agar diffusion assay against *SGM* using serial two-fold dilutions of *SGG* supernatant concentrated (SN 200X) or not (SN 1X) by ammonium sulfate precipitation. **B)** and **C)** Growth curves for the 12 gallocin A- resistant mutants in the presence or absence of gallocin A (THY

medium supplemented with 30% of *SGG* WT/Δ*blp* supernatant). This experiment was repeated three times independently.

Fig. S6: Mutations identified in key proteins of RSM mutants.

Amino acid sequence of WalK (A), WalR (B), and the aggregation promoting factor (C) in *SGM* WT. Putative domains, identified by BLAST, in these proteins are shown in red (HATPase_C: smart00387 ; REC: cd17614 ; Helix-turn-helix: pfam00486 ; LysM: cd00118 and Lysozyme-like: cd13925). The differences identified by sequencing in RSM mutants are indicated by arrows (STOP: apparition of a STOP codon, X → Y: substitution of the amino acid X by the amino acid Y). In case of substitution, the putative function of the amino acid (predicted by BLAST), if any, is indicated with an arrowhead.

Fig. S7: Putative structure of two-component bacteriocins

Structural models of mature forms of the two peptides composing the two-component bacteriocins ABP118, Brochocin C and Thermophilin 13 using ColabFold. The amino acid sequence following the first glycine doublet (in bold) was considered as the mature form of the peptides. Uniprot accession numbers: ABP118: Q8KWI0; Q8KWH9. Brochocin C: O85756; O85757. Thermophilin 13: O54454; O54455. All representations are colored with predicted lDDT from a score of 30% (red) to 100% (blue). The disulfide bond is visible in stick representation for all the peptides except BrcB.

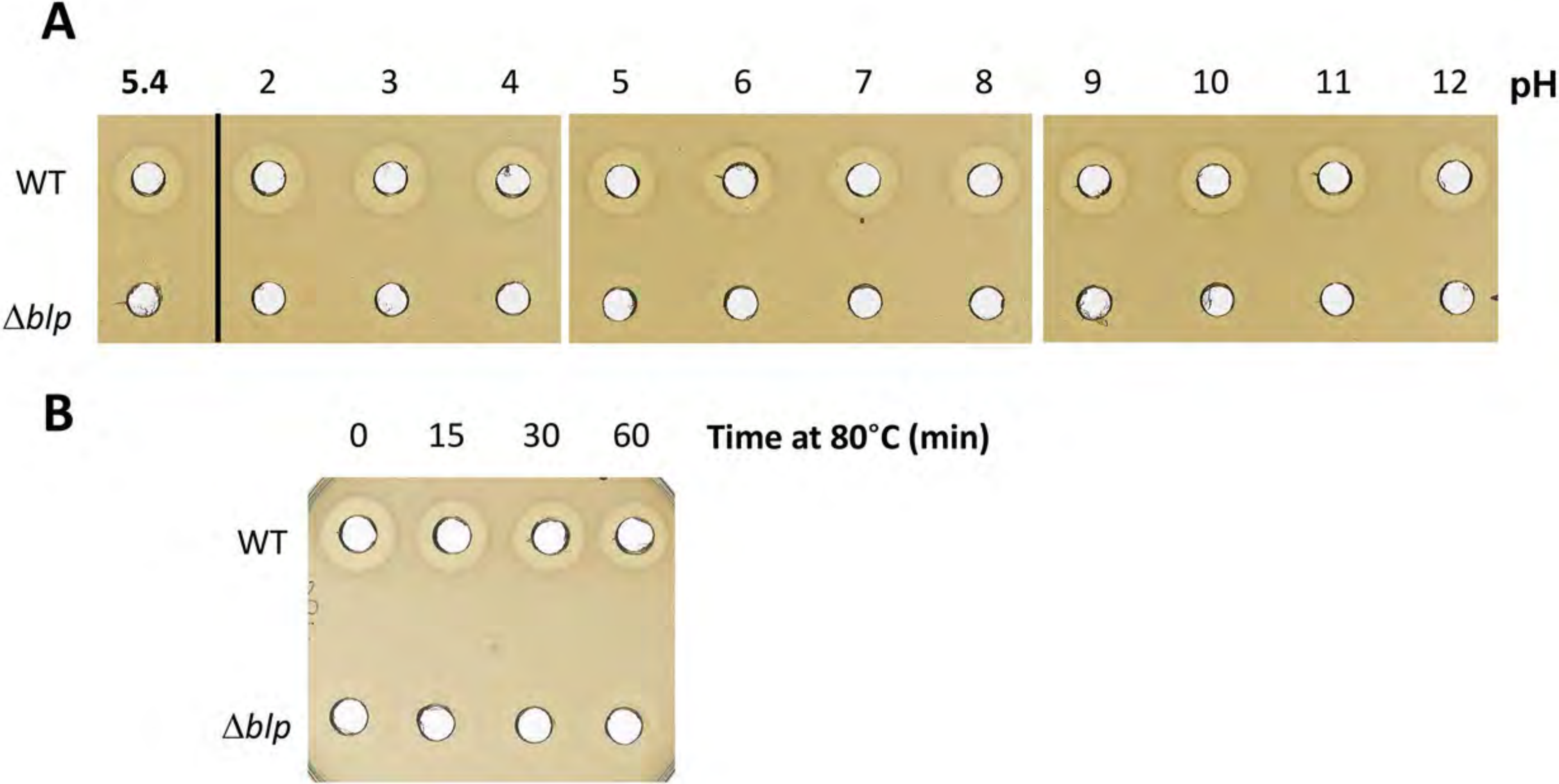
A
5.4
2
3
4
5
6
7
8
9
10
11
12
pH
WT
Δblp
B
0
15
30
60
Time at 80°C (min)
WT
Δblp

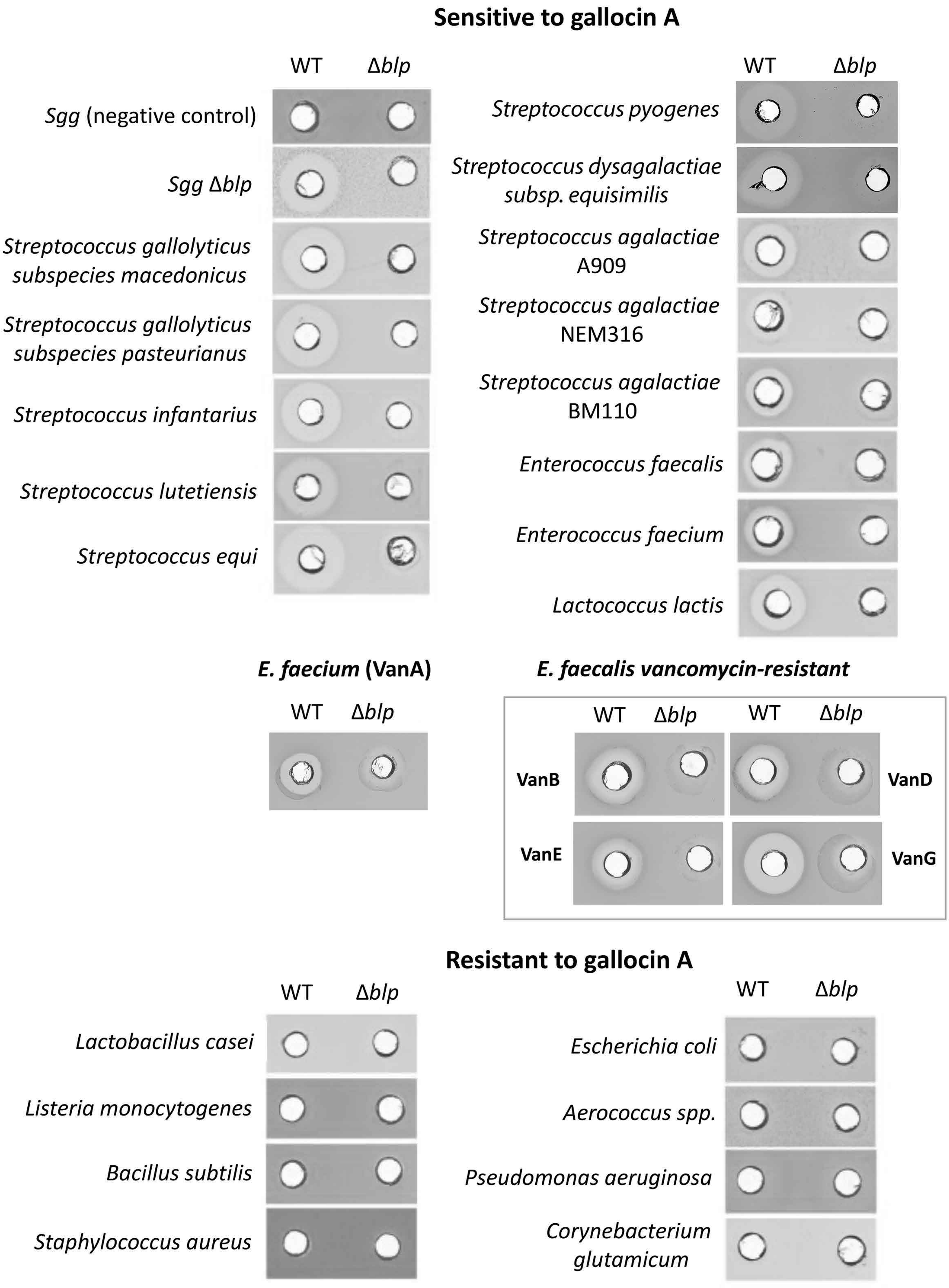
Sensitive to gallocin A
WT
Δblp
Sgg (negative control)
Sgg Δblp
Streptococcus gallolyticus subspecies macedonicus
Streptococcus gallolyticus subspecies pasteurianus
Streptococcus infantarius
Streptococcus lutetiensis
Streptococcus equi
Streptococcus pyogenes
Streptococcus dysagalactiae subsp. equisimilis
Streptococcus agalactiae A909
Streptococcus agalactiae NEM316
Streptococcus agalactiae BM110
Enterococcus faecalis
Enterococcus faecium
Lactococcus lactis
E. faecium (VanA)
E. faecalis vancomycin-resistant
VanB
VanD
VanE
VanG
Resistant to gallocin A
Lactobacillus casei
Listeria monocytogenes
Bacillus subtilis
Staphylococcus aureus
Escherichia coli
Aerococcus spp.
Pseudomonas aeruginosa
Corynebacterium glutamicum

GIIA2

$C_{241}H_{389}N_{65}O_{72}S_4$

MW: 5477.30 Da

YSKTDCLNAMITGIAGGIVAGGTGAGLVTLGVAGLPGAFVGAHIGAIGGGATCVGGMLFN

(Cys6-Cys53)

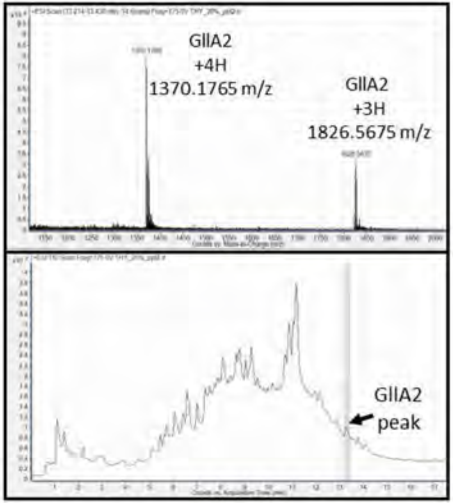

GIIA1

$C_{161}H_{270}N_{44}O_{50}S_4$

MW: 3750.38 Da

KGNMGSAIGGCIGGVLLAAATGPITGGGAAMICVASGISAYL

(Cys11-Cys33)

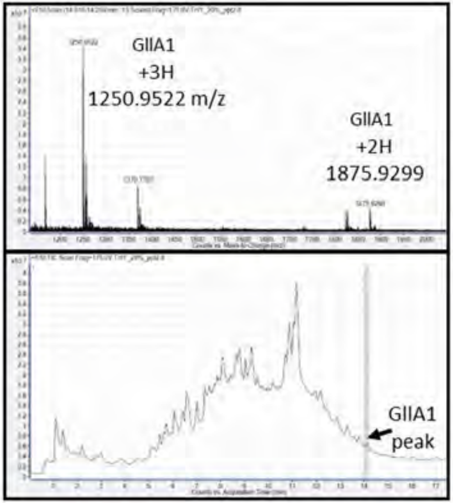

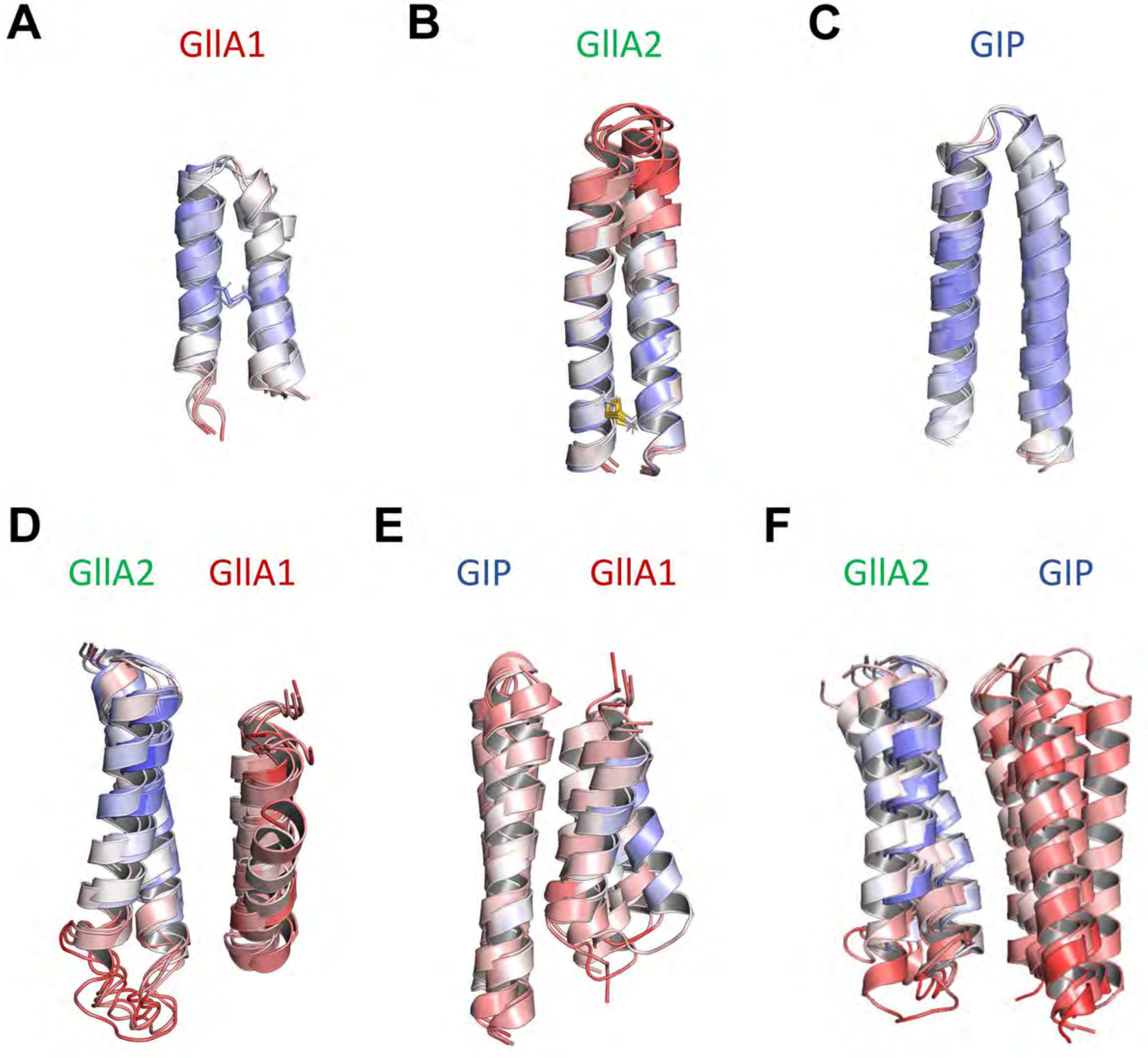

A
GllA1
B
GllA2
C
GIP
D
GllA2
GllA1
E
GIP
GllA1
F
GllA2
GIP

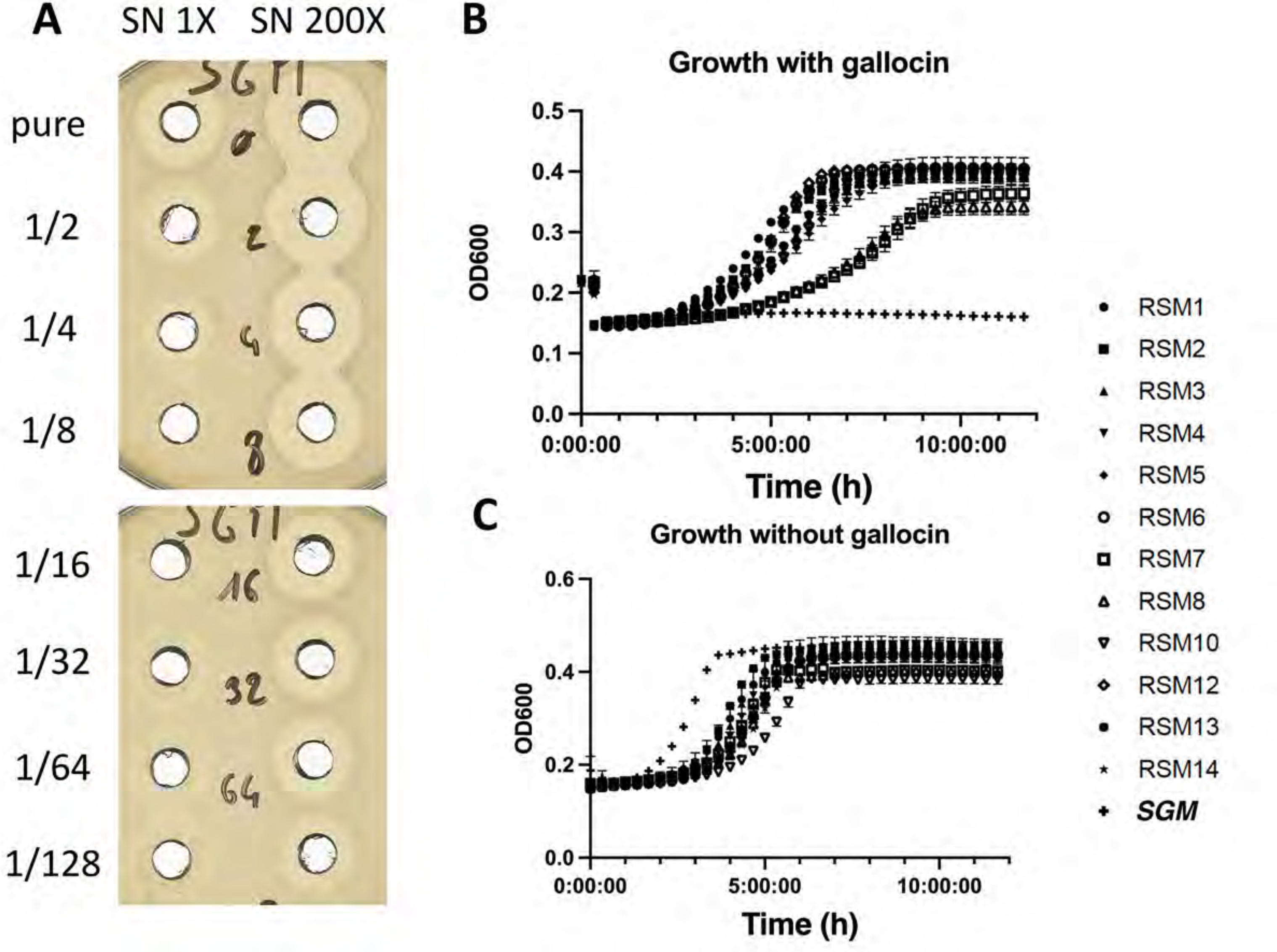
A
SN 1X
SN 200X
pure
1/2
1/4
1/8
1/16
1/32
1/64
1/128
B
Growth with gallocin
OD600
0.0
0.1
0.2
0.3
0.4
0.5
0:00:00
5:00:00
10:00:00
Time (h)
C
Growth without gallocin
OD600
0.0
0.2
0.4
0.6
0:00:00
5:00:00
10:00:00
Time (h)
RSM1
RSM2
RSM3
RSM4
RSM5
RSM6
RSM7
RSM8
RSM10
RSM12
RSM13
RSM14
SGM

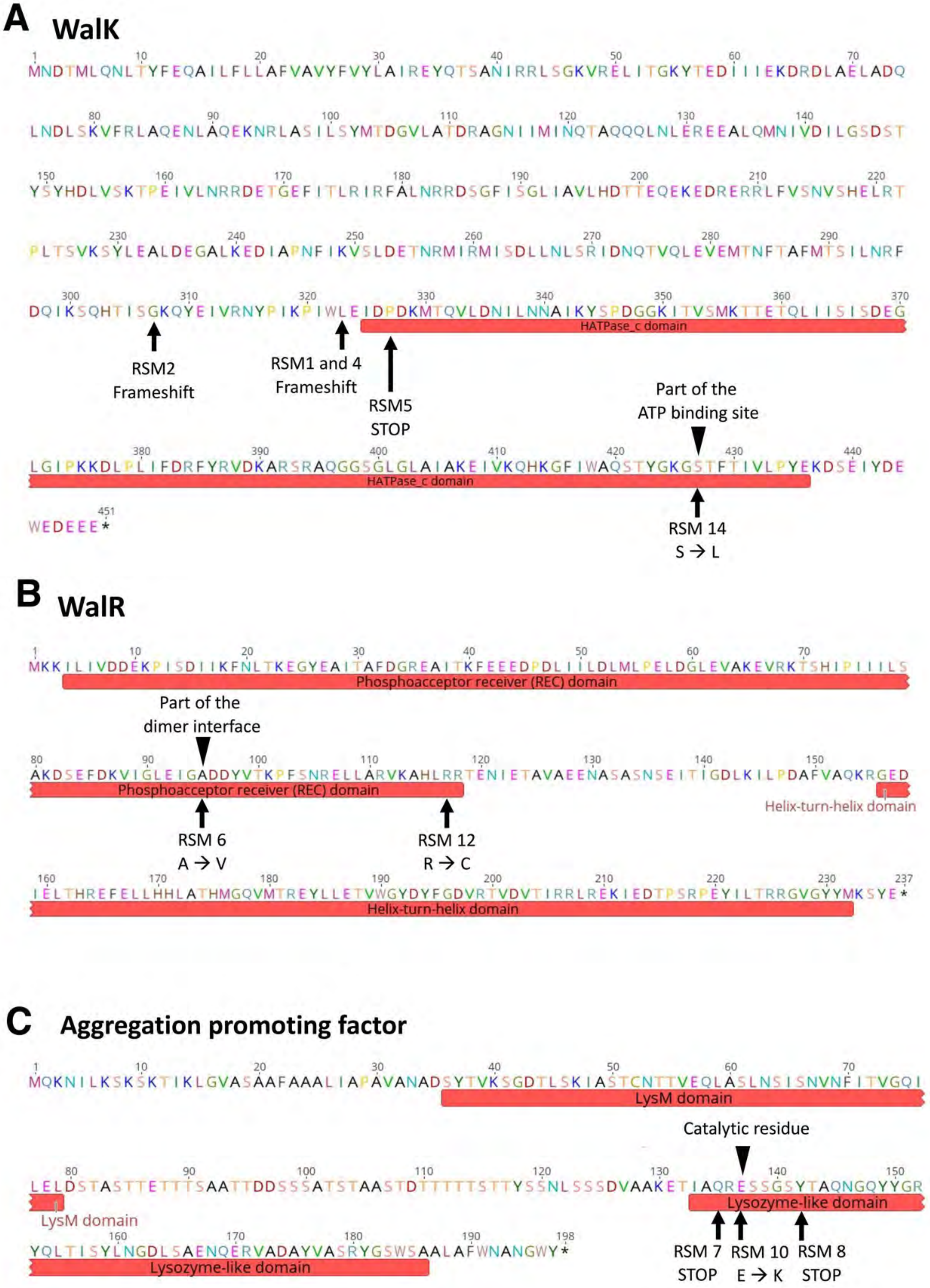
A
WalK
HATPase_c domain
RSM2
Frameshift
RSM1 and 4
Frameshift
RSM5
STOP
Part of the
ATP binding site
RSM 14
S → L
B
WalR
Phosphoacceptor receiver (REC) domain
Part of the
dimer interface
Helix-turn-helix domain
RSM 6
A → V
RSM 12
R → C
C
Aggregation promoting factor
LysM domain
Catalytic residue
Lysozyme-like domain
RSM 7
STOP
RSM 10
E → K
RSM 8
STOP

A

ABP118-α

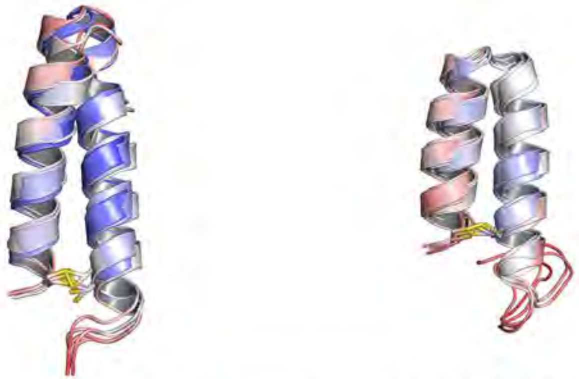

ABP118-β

ABP118-α: MMKEFTVLTECELAKVDGG**KRGPNCVGNFLGGLFAGAAAGVPLGPAGIVGGANLGMVGGALTCL**
ABP118-β: MKNLDKRFTIMTEDNLASVNGG**KNGYGGSGNRWVHCGAGIVGGALIGAIGGPWSAVAGGISGGFTSCR**

B

BrcA

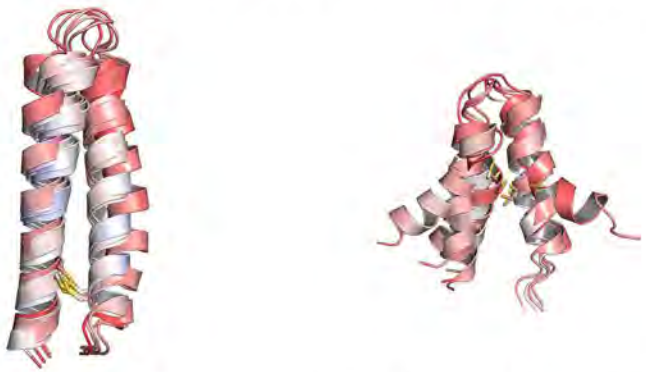

BrcB

BrcA: MHKVKKLNNQELQQIVGG**YSSKDCLKDIGKGIGAGTVAGAAGGGLAAGLGAIPGAFVGAHFGVIGGSAACIGGLLGN**
BrcB: MKKELLNKNEMSRIIGG**KINWGNVGGSCVGGAVIGGALGGLGGAGGGCITGAIGSIWDQW**

C

ThmA

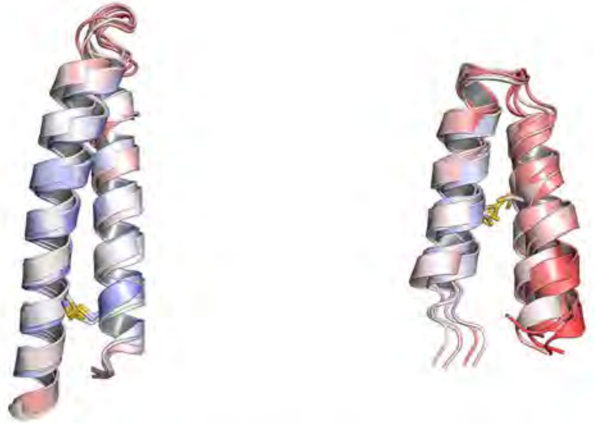

ThmB

ThmA:
MNTITICKFDVLDAELLSTVEGG**YSGKDCLKDMGGYALAGAGSGALWGAPAGGVGALPGAFVGAHVGAIAGGFACM GGMIGNKFN**
ThmB: MKQYNGFEVLHELDLANVTGG**QINWGSVVGHCIGGAIIGGAFSGGAAAGVGCLVGSGKAIINGL**
