# Supplementary material for: Gallocin A, an Atypical Two-Peptide Bacteriocin with Intramolecular Disulfide Bonds Required for Activity
Source: Microbiol Spectr. 2023 Mar 23;11(2):e05085-22. doi: 10.1128/spectrum.05085-22 (PMC10100652; doi:10.1128/spectrum.05085-22)
Supplement: Supplemental file 2 — Table S1 to S3. Download spectrum.05085-22-s0002.pdf, PDF file, 0.3 MB [file spectrum.05085-22-s0002.pdf]

Table S1: Single nucleotide polymorphisms detected in RSM mutants as compared to the control SGM WT

Strain	Position on *SGM* CIP105683T chromosome	Depth	Sequence in reference genome	Sequence in RSM	Type	Freebayes score	Strand balance	Fisher p-value	Frequency	CDS position	Effect type	Codon change	Gene name	Mutation type	Effect on protein	Protein size (in amino acids)
RSM-1	1609857	141	GCCAGAT	GCCAGACCAGAT	INDEL	4416,47	0,471	1	0,98	961_962insTCTGG	frameshift_variant	atc/aTCTGGtc	NPFEHBFA_01714		Leu323fs	450
RSM-1	1967146	171	G	T	SNV	5614,57	0,402	0,405882353	0,99	17C>A	missense_variant	gCc/gAc	NPFEHBFA_02107	MISSENSE	Ala6Asp	123
RSM-1	1987074	309	TGT	TGGT	INDEL	5417,64	0,428	0,246009502	0,54	1987075_1987076insG	intragenic_variant		NPFEHBFA_00018			
RSM-2	1609905	105	CGCTGATTGT	CGCTGATTGGCTGATTGT	INDEL	2980,49	0,447	0,4653761	0,90	910_911insCAATCAGC	frameshift_variant	aca/aCAATCAGCca	NPFEHBFA_01714		Gly307fs	450
RSM-3	1967146	191	G	T	SNV	6332,62	0,458	0,460732984	1,00	17C>A	missense_variant	gCc/gAc	NPFEHBFA_02107	MISSENSE	Ala6Asp	123
RSM-4	1609857	111	GCCAGATTGGTT	[illegible]CCAGATTGGTCCAGATTGGT	INDEL	2566,58	0,488	0,36118636	0,74	956_957insACCAATCTGG	frameshift_variant	aaa/aaACCAATCTGGa	NPFEHBFA_01714		Leu323fs	450
RSM-5	1609830	150	G	A	SNV	4961,5	0,493	1	1,00	994C>T	stop_gained	Cag/Tag	NPFEHBFA_01714	NONSENSE	Gln332*	450
RSM-5	2110152	194	C	T	SNV	6434,79	0,454	1	1,00	112G>A	missense_variant	Ggc/Agc	NPFEHBFA_02243	MISSENSE	Gly38Ser	139
RSM-6	1611243	145	G	A	SNV	4753,75	0,455	1	1,00	284C>T	missense_variant	gCa/gTa	NPFEHBFA_01715	MISSENSE	Ala95Val	236
RSM-7	59133	116	ATTTTTTGGTT	ATTTTTTTGGTT	INDEL	3769,47	0,47	1	0,99	112dupT	frameshift_variant	tgg/tTgg	NPFEHBFA_00076		Trp38fs	322
RSM-7	1665912	103	C	T	SNV	3443,77	0,422	1	0,99	1665912C>T	intragenic_variant		NPFEHBFA_00018			
RSM-7	2167995	122	G	A	SNV	3983,79	0,475	1	1,00	403C>T	stop_gained	Caa/Taa	NPFEHBFA_02313	NONSENSE	Gln135*	197
RSM-8	1028320	114	G	A	SNV	3821,48	0,421	1	1,00	107C>T	missense_variant	cCa/cTa	NPFEHBFA_01139	MISSENSE	Pro36Leu	87
RSM-8	2167972	163	G	T	SNV	5539,98	0,469	1	0,99	426C>A	stop_gained	taC/taA	NPFEHBFA_02313	NONSENSE	Tyr142*	197
RSM-9	970200	81	G	A	SNV	1543,39	0,438	0,012693076	0,59	970200G>A	intragenic_variant		NPFEHBFA_00018			
RSM-9	1608485	93	C	T	SNV	3076,42	0,467	1	0,99	1608485C>T	intragenic_variant		NPFEHBFA_00018			
RSM-10	890476	125	A	G	SNV	4177,47	0,464	1	1,00	829A>G	missense_variant	Act/Gct	NPFEHBFA_00992	MISSENSE	Thr277Ala	366
RSM-10	1111112	98	GAAAAAAATTG	GAAAAAATTG	INDEL	3313,57	0,449	1	1,00	1111118delA	intragenic_variant		NPFEHBFA_00018			
RSM-10	2167989	131	C	T	SNV	4416,49	0,489	1	1,00	409G>A	missense_variant	Gaa/Aaa	NPFEHBFA_02313	MISSENSE	Glu137Lys	197
RSM-11	987794	132	ACCGA	ACGA	INDEL	4361,88	0,473	1	0,99	438delC	frameshift_variant	acc/	NPFEHBFA_01100		Glu147fs	520
RSM-12	121316	138	T	G	SNV	4694,83	0,486	1	1,00	955T>G	stop_lost+splice_region_variant	Taa/Gaa	NPFEHBFA_00166	MISSENSE	Ter319Gluext*?	318
RSM-12	1611178	130	G	A	SNV	4376,41	0,408	1	1,00	349C>T	missense_variant	Cgt/Tgt	NPFEHBFA_01715	MISSENSE	Arg117Cys	236
RSM-13	404530	153	A	G	SNV	4984,05	0,49	1	1,00	314T>C	missense_variant	gTa/gCa	NPFEHBFA_00464	MISSENSE	Val105Ala	193
RSM-13	518528	144	C	G	SNV	4789,74	0,486	1	1,00	193C>G	missense_variant	Ccg/Gcg	NPFEHBFA_00589	MISSENSE	Pro65Ala	292
RSM-13	1847713	158	T	A	SNV	3277,13	0,408	0,867232731	0,62	1847713T>A	intragenic_variant		NPFEHBFA_00018			
RSM-14	1609544	106	G	A	SNV	3551,39	0,491	1	1,00	1280C>T	missense_variant	tCg/tTg	NPFEHBFA_01714	MISSENSE	Ser427Leu	450
RSM-14	2113956	123	C	T	SNV	4184,54	0,488	1	1,00	26G>A	missense_variant	gGa/gAa	NPFEHBFA_02247	MISSENSE	Gly9Glu	419

Number	Strains	Source
	***S. gallolyticus* strains**	
NEM 2431	*S. gallolyticus* subspecies *gallolyticus* UCN34	(Rusniok et al., 2010)
NEM 4838	UCN34 Δ*blp*	(Aymeric et al., 2018)
NEM 4694	UCN34 Δ*gllA1*	This work
NEM 4812	UCN34 Δ*gllA2*	This work
NEM 4988	UCN34 Δ*blpT* (*gallo_rs10370*)	This work
NEM 5097	UCN34 Δ*blpS*	(Proutiere *et al*., 2021)
NEM 1765	*S. gallolyticus* subspecies *macedonicus*	CIP 105683T
150507100801	*S. gallolyticus* subspecies *pasteurianus*	CNR collection (Cochin)
NEM 4801	UCN34 Δ*blp* pTCV P*tet-gip*	This work
NEM 4806	UCN34 Δ*blp* pTCV Ptet	This work
	**Heterologous expression of immunity protein**	
NEM 4828	*Lactococcus lactis* pTCV P*tet-gip*	This work
NEM 5667	Lactococcus lactis pTCV	This work
NEM4825	*Streptococcus agalactiae* A909 pTCV P*tet-gip*	This work
NEM3245	*Streptococcus agalactiae* A909 pTCV	This work
	**Strains tested for gallocin sensitivity**	
NEM 4825	*Streptococcus agalactiae* A909 pTCV Ptet-gip	Collection BBPG
NEM 1867	*Streptococcus infantarius*	CIP106105
NEM 640	*Streptococcus lutetiensis*	Collection BBPG
NEM 739	*Streptococcus equi*	Collection BBPG
NEM 2526	*S. agalactiae* A909	Collection BBPG
NEM 3525	*S. agalactiae* NEM316	Collection BBPG
NEM 2312	*S. agalactiae* BM110	Collection BBPG
NEM 409	*Enterococcus faecalis*	Collection BBPG
NEM 489	*Enterococcus faecium*	Collection BBPG
NEM 4906	*Lactococcus lactis*	Collection BBPG
NEM 4703	*Lactobacillus casei*	Collection BBPG
NEM 140	*Listeria monocytogenes* F6953	Collection BBPG
NEM 466	*Bacillus subtilis*	Collection BBPG
NEM 416	*Staphylococcus aureus* RN4220	Collection BBPG
NEM 453	*Escherichia coli*	Collection BBPG
NEM 761	*Aerococcus spp.*	Collection BBPG
NEM 486	*Pseudomonas aeruginosa*	Collection BBPG
NEM 602	*Corynebacterium glutamicum*	Collection BBPG
	**Vancomycin resistant Enterrococcus**	
CIP 103510	*E. faecium* (VanA)	Collection Institut Pasteur
CIP 111159	*E. faecalis* (VanB)	Collection Institut Pasteur
CIP 111253	*E. faecalis* (VanD)	Collection Institut Pasteur
CIP 111106	*E. faecalis* (VanE)	Collection Institut Pasteur
CIP 111107	*E. faecalis* (VanG)	Collection Institut Pasteur
	**Competition expriment**	
NEM 2829	*Enterococcus faecalis* OG1RF	Collection BBPG
	***Streptococcus gallolyticus* subspecies *macedonicus* mutants resistant to gallocin (RSM)**	
NEM 5627	RSM1	This work
NEM 5628	RSM2	This work
NEM 5629	RSM3	This work
NEM 5630	RSM4	This work
NEM 5631	RSM5	This work
NEM 5632	RSM6	This work
NEM 5633	RSM7	This work
NEM 5634	RSM8	This work
NEM 5636	RSM10	This work
NEM 5638	RSM12	This work
NEM 5639	RSM13	This work
NEM 5640	RSM14	This work

- Rusniok C, Couve E, Da Cunha V, El Gana R, Zidane N, Bouchier C, Poyart C, Leclercq R, Trieu-Cuot P, Glaser P. 2010. Genome Sequence of Streptococcus gallolyticus: Insights into Its Adaptation to the Bovine Rumen and Its Ability To Cause Endocarditis. 8. J Bacteriol 192:2266–2276. https://journals.asm.org/doi/10.1128/JB.01659-09.
- Aymeric L, Donnadieu F, Mulet C, Du Merle L, Nigro G, Saffarian A, Bérard M, Poyart C, Robine S, Regnault B, Trieu-Cuot P, Sansonetti PJ, Dramsi S. 2018. Colorectal cancer specific conditions promote Streptococcus gallolyticus gut colonization. Proc Natl Acad Sci U S A 115:E283–E291. https://doi.org/10.1073/pnas.1715112115.
- Proutière A, Du Merle L, Périchon B, Varet H, Gominet M, Trieu-Cuot P, Dramsi S. 2021. Characterization of a four-component regulatory system controlling bacteriocin production in Streptococcus gallolyticus. mBio 12:e03187-20. https://doi.org/10.1128/mBio.03187-20.

Table S3: List of primers used in this study

Primers	Sequence (5'-3')
**Deletions**	
gllA1	TTCT**GAATTC**GAAACTAGAACTATTGTGCC
	TTCTATAAGTATGCTGAAATACTCTCTCCTTATAAA
	TTTATAAGGAGAGAGTATTTCAGCATACTTATAGA
	TTCT**GGATCC**CAGGCAATATTATTGCCAT
gllA2	TTCT**GAATTC**TAATGCGGGAGTTTGCCT
	ACTCTCTCCTTATAAAATTATTGAATACCTCCCAATAA
	TTATTGGGAGGTATTCAATAATTTTATAAGGAGAGAGT
	TTCT**GGATCC**CAGGCAATATTATTGCCA
blpT (*gallo_RS10370*)	TTCT**GAATTC**AATCCCAGATAGACCGCC
	GCAACTGTTTTATCAATGGGCAGAGGAAAAGTAGCA
	TGCTACTTTTCCTCTGCCCATTGATAAAACAGTTGC
	TTCT**GGATCC**CGACAGACGGTATGTTAG
**Overexpression**	
gip	TTCT**GGATCC**ATTGGGAGGTATTCAAATGATTATAAAATATAG
	TTCT**CTGCAG**CAATAGTAATACATTAT

Restriction sites are indicated in bold
